# Genomics and Epigenomics in Parathyroid Neoplasia: from Bench to Surgical Pathology Practice

**DOI:** 10.1007/s12022-020-09656-9

**Published:** 2020-12-02

**Authors:** C. Christofer Juhlin, Lori A. Erickson

**Affiliations:** 1 Department of Oncology-Pathology, Karolinska Institutet, Solna, Sweden; 2 Department of Pathology and Cytology, Karolinska University Hospital, Stockholm, Sweden; 3 Department of Laboratory Medicine and Pathology, Mayo Clinic, Rochester, MN USA

**Keywords:** Parathyroid tumors, Primary hyperparathyroidism, Pathology, Genetics, Review

## Abstract

The majority of parathyroid disease encountered in routine practice is due to single parathyroid adenoma, of which the majority arise as sporadic tumors. This is usually a straightforward diagnosis in endocrine pathology when in the appropriate clinical setting, although subsets of cases will exhibit atypical histological features that may warrant additional immunohistochemical and genetic analyses to estimate the malignant potential. Parathyroid carcinomas on the other hand, are bona fide malignant tumors characterized by their unequivocal invasion demonstrated through routine histology or metastasis. The ultimate endpoint for any molecular marker discovered through laboratory investigations is to be introduced in clinical routine practice and guide the surgical pathologist in terms of diagnostics and prognostication. For parathyroid tumors, the two main diagnostic challenges include the distinction between parathyroid adenoma and parathyroid carcinoma, as well as the pinpointing of hereditable disease for familial screening purposes. While numerous markers on genetic, epigenetic, and protein levels have been proposed as discriminative in these aspects, this review aims to condense the scientific coverage of these enigmatic topics and to propose a focused surgical pathology approach to the subject.

## Introduction

### General Background

In the clinical setting, the tumor responsible for primary hyperparathyroidism (PHPT) is usually a parathyroid adenoma. The vast majority of parathyroid adenomas are functioning due to an altered set point in terms of calcium sensing mechanisms, and the ensuing parathyroid hormone (PTH) secretion leads to hypercalcemia that may cause diverse symptoms in the afflicted patient [1]. However, the majority of parathyroid adenomas are identified through serum calcium screening. The peak incidence is among 50–60-year-old individuals, and the female to male ratio increases with increased age at diagnosis, reaching 5:1 among patients > 75 years of age [2]. The treatment is surgical, and cure rates at tertiary centers are usually high [3]. Most cases are preoperatively localized using combinations of various imaging techniques, such as neck ultrasound, single-photon emission computed tomography (SPECT/CT), and/or technetium (99mTc) sestamibi scintigraphy, and the endocrine surgeon can thus plan for a focused parathyroidectomy [4, 5]. Although the bulk of PHPT cases are sporadic tumors arising through the somatic acquisition of genetic aberrancies in driver genes, approximately 5% of cases are associated with familial disease. If a familial syndrome is suspected, a four-gland exploration with subtotal parathyroidectomy or total parathyroidectomy with intramuscular reimplantation is often the preferred strategy, as these patients may develop multiglandular, metachronous disease—and some also carry a risk of developing parathyroid carcinoma [6, 7].

**Fig. 1:**
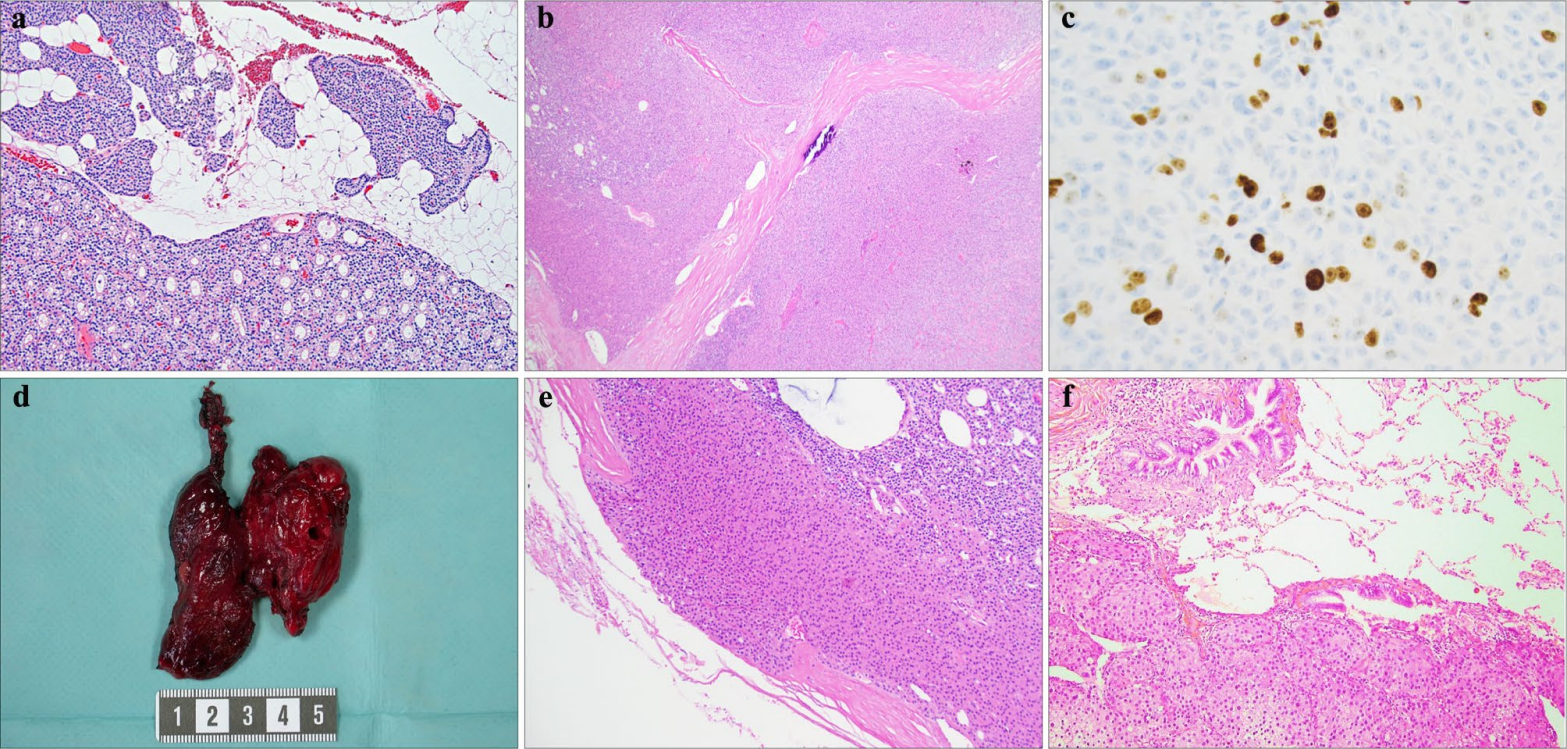
Histological characteristics of parathyroid tumors. All stainings are routine hematoxylin–eosin (H&E) unless otherwise specified. **a** Parathyroid adenoma (PA) characterized by chief cells in acinar formations with a clear reduction in stromal fat content. Note the adjacent normal parathyroid rim in the upper part of the image, with palisading nuclei and a generous admixture of adipose tissue. Magnification × 100. **b** Oxyphilic parathyroid tumor with high cellularity and fibrous bands. This tumor was clearly enlarged (2 g) and also displayed equivocal relations to the surrounding capsule. The final diagnosis was an atypical parathyroid tumor. Magnification × 40. **c** Parathyroid carcinoma (PC) immunohistochemically stained for Ki-67, displaying a marked increase in proliferative activity (11%). Magnification × 400. **d** Gross image of a resected PC, adherent to the left thyroid lobe. This tumor was found to infiltrate the thyroid parenchyma upon histological investigation. **e** PC displaying capsular invasion. Note the mushroom-like protrusion. **f** PC metastatic to lung. Magnification × 100

From a histopathological perspective, parathyroid adenomas are usually well-circumscribed tumors composed of chief cells arranged in micro-acinar or palisading formations (Fig. 1a) [8]. Subsets of cells can exhibit hyperchromatic nuclei with nuclear atypia, and multinucleated tumor cells are sometimes observed. Mitotic figures can be seen in parathyroid adenoma and parathyroid carcinoma. The stromal fat content is reduced compared to normal gland histology, and a rim of normal appearing (although suppressed) parathyroid tissue can usually be seen, particularly in smaller parathyroid adenomas and less commonly seen in larger adenomas (Fig. 1a). What appears to be a rim of normal-appearing parathyroid tissue cannot be used to differentiate parathyroid adenoma from multiglandular disease, as up to 10% of parathyroid “hyperplasia” (multiglandular disease) may have what appear to be rims of normal parathyroid. Moreover, as intraoperative frozen section analyses not always are effective in distinguishing single parathyroid adenomas from multiglandular involvement, intraoperative PTH assays are probably more reliable in this context [9]. In order to diagnose a parathyroid adenoma, there must be no signs of malignant behavior, such as vascular or perineural invasion. Regarding their immunophenotype, most parathyroid adenomas are positive for chromogranin A, PTH, and GATA3. Numerous histological variants have been described, of which oncocytic parathyroid adenomas are the most common, followed by unusual variants such as parathyroid lipoadenomas and water-clear cell adenomas [8, 10–12]. From a clinical perspective, oncocytic features associate to larger tumor size. Oncocytic parathyroid tumors are functional but may not have as elevated serum calcium or parathyroid hormone levels as comparable conventional parathyroid adenomas. Lipoadenomas might possibly correlate to the presence of arterial hypertension—suggesting that these subclassifications might disclose clinical considerations of importance [11, 13].

Subsets of parathyroid tumors exhibit atypical features usually observed in carcinomas, but yet lacking unequivocal criteria of malignancy such as invasive growth into periparathyroidal tissues (thyroid gland or soft tissues), lymphovascular or perineural invasion, regional and/or distant metastases. These features may include a trabecular growth pattern, fibrotic bands, tumor cells within the capsule, increased mitotic/proliferative activity, and nuclear atypia with macronucleoli (Fig. 1b, c) [8, 14–16]. Parathyroid lesions with these features are termed “atypical parathyroid tumors” and could be considered tumors with unknown malignant potential—even though the majority of these cases will behave benign in a clinical context, with few recurrences following parathyroidectomy [17, 18]. Due to the fact that small subsets of atypical tumors will recur as metastatic parathyroid carcinomas, numerous studies have tried to highlight the malignant potential of this tumor subgroup using various combinations of histology and molecular markers, which are discussed in detail below.

Parathyroid carcinoma is the malignant form of PHPT. Quite rare, accounting for < 1% of PHPT, parathyroid carcinoma is an entity which from an endocrine pathology perspective always is feared, often discussed, but rarely diagnosed (personal observations) [8, 19]. Unlike parathyroid adenomas, which are more common in women than in men, parathyroid carcinomas affect woman and men equally. Clues to a parathyroid carcinoma diagnosis can be obtained preoperatively, as these tumors often are larger than adenomas, may be clinically palpable, and usually are associated with high serum calcium levels (often > 13.5 mg/dl) with patients exhibiting various symptoms related to hypercalcemia, such as nephrolithiasis and bone disease [20, 21]. Perioperative findings of the tumor being adherent to adjacent structures can also be information of value when assessing these rare lesions, and for this reason, surgeons usually opt in for an en bloc resection of the afflicted ipsilateral thyroid lobe when suspecting parathyroid carcinoma (Fig. 1d) [21]. However, one must be cautious in evaluating a parathyroid that may be adhesed to adjacent structures such as the thyroid gland, as parathyroid adenomas may also be firmly adhesed to this organ. The difference of course is that parathyroid adenomas are not invasive of the thyroid while parathyroid carcinomas may be invasive of the adjacent thyroid. Parathyroid carcinomas exhibit local invasive behavior and may spread locally to adjacent structures and later on to distant sites (Fig. 1e, f) [8]. Chemotherapy and/or radiotherapy are largely ineffective, and the 10-year survival rate is around 50–70% [22], with death often due to hypercalcemia. Morbidity due to complications following repetitive neck surgery (hypoparathyroidism, recurrent nerve palsy) is also high [23].

The differentiation of parathyroid carcinoma from other carcinomas in the neck is usually straightforward. Parathyroid carcinomas are neuroendocrine tumors and generally positive for chromogranin A. Other neuroendocrine tumors in the neck such as medullary thyroid carcinoma will also shows staining for chromogranin A, but medullary thyroid carcinomas are usually positive for thyroid transcription factor 1 while negative for parathyroid hormone. Calcitonin is usually positive in medullary thyroid carcinoma and usually negative in parathyroid carcinoma; however, unusual staining patterns can be seen such an exceptional case of parathyroid carcinoma positive for calcitonin and calcitonin gene related peptide [24]. Moreover, practicing pathologist should also be aware that medullary thyroid carcinomas can express PAX8 in a clone-dependent manner, in which absence of immunoreactivity is noted when monoclonal PAX8 antibodies are applied, as opposed to positive staining using polyclonal antibodies [25, 26]. As parathyroid tissue might stain positive for polyclonal PAX8 antibodies, we recommend a distinguishing panel of PTH and TTF1 when assessing these differentials [27].

Although a plethora of epigenetic and genetic aberrancies have been identified in parathyroid tumors, few markers have paved their way into clinical routine practice. In this review, we focus on two main clinical predicaments concerning parathyroid tumors, namely, (1) the identification of tumors associated to various underlying syndromes of importance for genetic counseling, and (2) the distinction between benign and malignant tumors to triage each patient to correct follow-up and treatment algorithms. In the following sections, we highlight molecular markers of importance that facilitate these diagnostic quandaries, and discuss their potential as discriminative clinical markers of value to the surgical pathologist.

### Familial PHPT: Underlying Causes

Much of what we today know about mutational driver events in parathyroid tumorigenesis stems from earlier work in kindreds with familial PHPT, long before the appearance of next-generation sequencing techniques. By genetic linkage analyses of family members with autosomal dominant PHTP, candidate gene loci segregating with disease-afflicted individuals were identified, followed by identification of mutational events by cumbersome Sanger sequencing of a large number of candidate genes within these regions. By this methodology, germline mutations of the *MEN1 *(*multiple endocrine neoplasia type 1*), *RET* (*rearranged during transfection*), and *CDC73* (*cell division cycle protein 73*) (originally entitled *hyperparathyroidism type 2*, *HRPT2*) genes as events underlying the development of multiple endocrine neoplasia type 1 (MEN1), multiple endocrine neoplasia type 2A (MEN2A), and hyperparathyroidism-jaw tumor (HPT-JT) syndromes, respectively (Table 1) [28–36]. These three conditions exhibit high prevalence of PHPT, occurring in approximately 90% of MEN1 kindred, 20–30% of MEN2A kindred, and in 80% of HTP-JT kindred [8]. In MEN1 patients, PHPT is the most common disease manifestation, followed by pituitary tumors (30–40% of patients), duodenal and pancreatic neuroendocrine tumors (most often gastrinoma, insulinoma and/or glucagonoma, 40% of patients), and adrenocortical lesions (20–45% of patients) [8, 37]. In MEN2A, most patients (> 90%) develop medullary thyroid carcinoma and pheochromocytoma (50%), whereas PHPT is more uncommon, occurring in 15–30% of patients [8, 38]. For HPT-JT kindred, 70–80% develop PHPT, whereas approximately 10% of patients also develop ossifying fibromas of the mandible or maxilla [8, 39]. PHPT in MEN1 usually presents as multiglandular disease that may develop in synchronous or metachronous settings and may be asymmetric. For the MEN2A syndrome, the PHPT may present as multiglandular or single gland disease. There are exceedingly few reports of unequivocal parathyroid carcinomas arising in MEN1 or MEN2A kindred [40–42]. The hyperparathyroidism in HPT-JT syndrome is usually associated with a parathyroid adenoma; however, 15–40% of patients carrying *CDC73* mutations or gene deletions will develop parathyroid carcinoma [8, 43, 44].

**Table 1:** Overview of general constitutional and somatic genetic aberrations in parathyroid adenomas and carcinomas

	*MEN1 *mut.	*RET *mut.	*CDC73 *mut.	*CaSR *mut.	*GCM2 *mut.	*CDKi *family mut.	*PTH-CCND1* inversion	*CTNNB1 *mut.	*EZH2 *mut.	mtDNA mut.	*TP53 *mut.	*TERTp* mut.	*RIZ1 *meth.	*RASSF1A *meth.	*APC *meth.
Familial forms															
MEN1	**1**	-	-	-	-	-	-	-	-	-	-	-	-	-	-
MEN2a	-	**1**	-	-	-	-	-	-	-	-	-	-	-	-	-
HPT-JT	-	-	**1**	-	-	-	-	-	-	-	-	-	-	-	-
FHH1	-	-	-	**1**	-	-	-	-	-	-	-	-	-	-	-
FIHP	**2**	-	**3**	**3**	**3**	-	-	-	-	-	-	-	-	-	-
MEN1 variants	-	-	-	-	-	**1**	-	-	-	-	-	-	-	-	-
Sporadic PHPT															
PA	***3***	-	***4***	-	-	***4***	***4***	***4***	***4***	***2***	-	-	***3***	***1***	***2***
PC	***4***	-	***2***	-	-	-	-	-	-	-	***4***	***4***	-	-	***2***

*mut* mutation, *meth* aberrant promoter methylation, *CDKi* cyclin-dependent kinase inhibitor, *mtDNA* mitochondrial DNA, *TERTp* TERT promoter, *PA* parathyroid adenoma, *PC* parathyroid carcinoma

**Germline**

***Somatic***

(1) Almost always present (81–100%)

(2) frequently present (41–80%)

(3) sometimes present (11–40%)

(4) rarely present (≤ 10%)

-; not reported

Apart from these three syndromes, hyperparathyroidism is also seen as the sole feature in familial isolated hyperparathyroidism (FIHP). These families often, but not always, present with germline mutations in either *MEN1*, *CDC73*, or the *calcium sensing receptor* (*CaSR*) gene, of which the latter is also associated to the development of familial hypocalciuric hypercalcemia type 1 (FHH1) [45–50]. The reason why some patients with germline mutations develop full-blown MEN1, HPT-JT, and FHH1 syndromes, while others develop FIHP, is not clearly understood—as there is no apparent genotype to phenotype correlation in terms of mutation types and exon localization. Recently, germline activating *glial cells missing transcription factor 2* (*GCM2*) gene mutations were also coupled to FIHP, adding yet another candidate to the growing palette of genes underlying the development of familial hyperparathyroidism [51]. Moreover, germline inactivating mutations in cyclin-dependent kinase inhibitor (CDKI) genes have been found in rare families with MEN1-like syndromes (with mutations in either *CDKN1A*, *CDKN2B*, or *CDKN2C*) or the MEN4 syndrome, a phenotypic MEN1 syndrome characterized by mutations in *CDKN1B* [50, 52, 53].

### Somatic Genetics in Parathyroid Adenomas

**Fig. 2:**
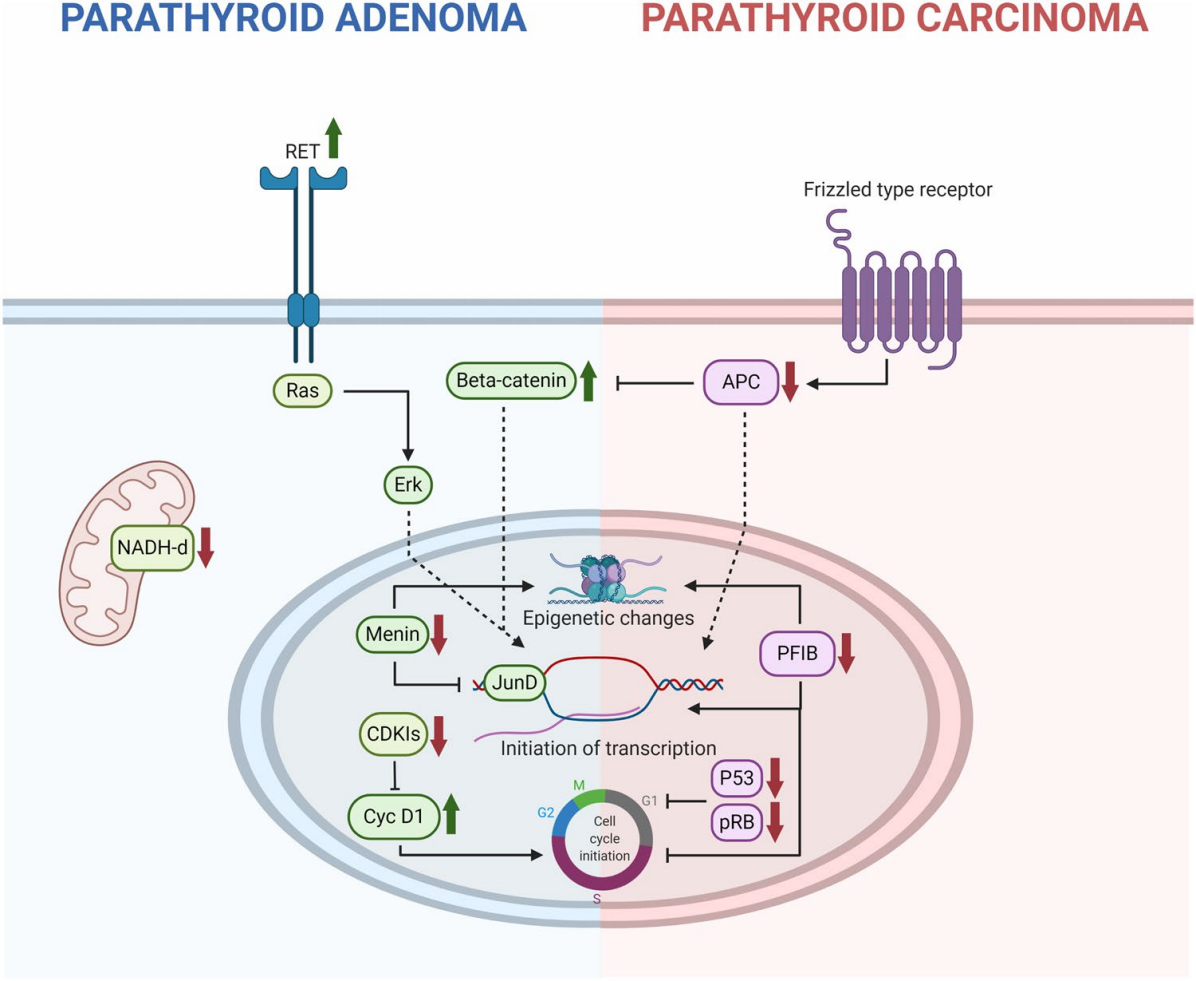
Schematic presentation of common molecular genetic aberrancies in parathyroid adenoma (PA; left) and carcinoma (PC; right). Green arrows represent activating events (such as activating mutations and/or protein overexpression) while red arrows depict deleterious events (inactivating mutations, downregulation or epigenetic silencing). Germline *RET* oncogenic mutations lead to activation of an intracellular Erk-RAS cascade followed by initiation of gene transcription and increased proliferation. Similarly, germline and somatic *MEN1* inactivating mutations affect the regulation of the JunD transcription regulator. Moreover, germline and somatic *CDC73* mutations affect epigenetic, transcriptional and cell cycle programs, underlying the development of both PAs and PCs. Additional mechanisms underlying the development of small subsets of PAs include mutational or epigenetic silencing of various cyclin-dependent kinase inhibitors (CDKIs) and up-regulation of cyclin D1 through a chromosomal inversion or via additional unknown mechanisms. Activating mutations in mitochondrial DNA (particularly *NADH* dehydrogenase gene family orthologs) and *CTNNB1* (encoding beta-catenin) could underly the development of PA cases, although their specific roles as driver gene events are debated. For PCs, inactivating *CDC73* gene mutations or promoter hypermethylation leads to the downregulation of parafibromin (PFIB), which regulates epigenetic, transcriptional and cell cycle programs. Depletion of additional cell cycle regulators (P53 and pRB) is also a recurrent theme in PC. Moreover, APC is in its normal state a negative regulator of the Wnt pathway, thereby inhibiting beta-catenin in the absence of upstream signaling from Frizzled type of membrane-bound receptors. Hypermethylation of the APC promoter has been demonstrated in PCs, thereby enhancing the Wnt pathway output—but APC also has DNA-binding capacity on its own and could possibly act independently from beta-catenin. Created with BioRender.com.

Given the identification of the *MEN1*, *RET*, and *CDC73* gene aberrancies as main responsible for the development of familial PHPT, numerous studies followed in which the involvement of these genes were assessed in sporadic parathyroid tumors. Approximately 25–40% of all sporadic parathyroid adenomas harbor LOH of the *MEN1* gene locus at 11q13, and half of these cases also exhibit an inactivating *MEN1* mutation of the remaining allele (Table 1, Fig. 2) [54, 55]. Interestingly, an association between somatic *MEN1* mutations and mild PHPT symptoms has been observed, possibly arguing for early-stage events in parathyroid tumorigenesis [55]. Moreover, while somatic *CDC73* gene mutations have been reported in small subsets of sporadic parathyroid adenomas, no reports on somatic *RET* gene mutations in parathyroid adenoma have been noted [35, 56, 57].

Moreover, mutational and/or epigenetic silencing of other genes predisposing for FIHP and MEN1-like syndromes have also been detected in small subsets of apparent sporadic parathyroid adenomas, including *CDKN1A*, *CDKN1B*, *CDKN2A*, *CDKN2B*, *CDKN2C*, and *GCM2* [53, 58–60]. Of particular interest, *CDKN1B* encodes p27 and down-regulation of p27 has been described in PAs, both on the RNA- and protein level [61–63]. Moreover, *CDKN1B* mutations have also been functionally linked to the development of parathyroid tumors, thereby solidifying the role of aberrant cell cycle regulation in the development of parathyroid adenomas [64]. Continuing on the cell cycle topic, a recurrent chromosomal inversion involving the peri-centromeric portion of chromosome 11 has been observed in exceedingly small subsets of sporadic parathyroid adenomas. This rearrangement causes the juxtaposition of the *PTH* 5′ regulatory region to the *CCND1* oncogene coding region, causing a constitutively expression of the *CCND1* corresponding protein cyclin D1. Although rare in sporadic parathyroid adenoma, overexpression of cyclin D1 is a common event, and therefore, other mechanisms apart from rare chromosomal inversions involving *CCND1* or mutations in cyclin D1-regulating CDKIs are expected to play a role. Instead, promoter hypermethylation and down-regulation of various CDKIs could probably explain the commonly observed cyclin D1 upregulation in parathyroid adenomas (Fig. 2) [65].

Apart from the discovery of somatic alterations in established parathyroid adenoma susceptibility genes discussed above, the advent of next-generation sequencing techniques has led to the discovery of additional gene mutations of possible importance to parathyroid adenoma development. By whole-exome sequencing of parathyroid adenomas, an activating missense mutation in the methyltransferase gene *enhancer of zeste homolog 2* (*EZH2*) was detected in one out of 8 adenomas interrogated, and additional targeted sequencing of 185 adenomas revealed one additional case with the same mutation [66]. The *EZH2* gene is an epigenetic regulator of chromatin accessibility with an association to tumorigenesis in general, thereby providing additional validity of this gene constituting a possible contributor of parathyroid adenoma development—which was also verified using functional experiments in a parathyroid cell line [67, 68]. Since then, additional whole-exome sequencing studies have corroborated low frequencies of *EZH2* mutations in sporadic adenomas [69].

Additional mutational events occurring in low frequencies of parathyroid adenomas include activating *CTNNB1* mutations, of which some have been reported as homozygous in small numbers [70], although not reproduced by others [71–73]. *CTNNB1* encodes beta-catenin, a central onco-protein regulating the Wingless type (Wnt) pathway, and data suggests that nuclear accumulation of beta-catenin could be an important player in the development of parathyroid adenomas—either through activating mutations or through aberrantly expressed Wnt co-receptors (Fig. 2) [74].

On the epigenetic level, apart from hypermethylation of CDKIs mentioned above, aberrant methylation has also been reported for numerous tumor-related genes, including *WT1*, *SFRP1*, *SFRP2*, *SFRP4*, *RIZ1*, *APC*, and *RASSF1A* [59, 65, 75–77]. Most notably, *RASSF1A* hypermethylation was strongly associated to down-regulation on the mRNA level in virtually all parathyroid adenomas, thereby constituting one of the most commonly known genetic aberrancies in this disease (Table 1) [75]. Moreover, on a global level, adenomas seem to exhibit similar levels of methylation as non-tumorous parathyroid tissues, suggesting that epigenetic dysregulations are driven by gene-specific events and not due to a general hypo- or hypermethylation pattern [75].

Regarding specific parathyroid adenoma subtypes, there is also an established correlation between oncocytic parathyroid adenomas and somatic mutations in genes encoded by mitochondrial DNA (mtDNA), especially mitochondrial respiratory chain complex genes *NADH dehydrogenase 1*, *4*, and *5* (*ND1*, *ND4*, and *ND5*) (Table 1, Fig. 2). As oncocytic tumors in general exhibit prominent amounts of mitochondria, the association is intriguing [78]. However, as no recurrent mutations were observed, these findings mandate functional verification before a true driver status could be assigned to any of these alterations.

### Somatic Genetics in Parathyroid Carcinomas

Somatic *CDC73* gene mutations are the most frequent somatic alteration in parathyroid carcinoma (Table 1, Fig. 2) [79–82]. These mutations are in general disruptive due to premature truncations or frameshift alterations, alternatively the mutations are of missense nature in conserved regions encoding the nuclear localization signals (NLSs) or the human polymerase–associated factor 1 (hPAF1) complex of the corresponding protein product, termed parafibromin. Apart from mutations, LOH encompassing the *CDC73* gene locus and aberrant *CDC73* promoter methylation have also been reported as somatic events in parathyroid carcinoma [83–85].

Parafibromin is a member of the hPAF regulatory complex, a key transcriptional unit that interacts with RNA polymerase II and facilitates transcriptional activity due to histone-modifying and chromatin remodeling processes [86]. Parafibromin is associated with tumor-suppressive properties, as (a) the majority of *CDC73* germline mutations in HPT-JT and FIHP kindred as well as the bulk of somatic mutations in sporadic parathyroid carcinomas are disruptive [35, 43, 79–81, 87–89], (b) the majority of tumors with *CDC73* mutations exhibit loss of parafibromin expression [57, 89–95], and (c) functional experiments with *CDC73* plasmids support an anti-proliferative effect of the wild-type protein [96]. Indeed, parafibromin has been found to regulate cyclin D1 levels, exhibit pro-apoptotic effects, regulate the Wnt pathway through interactions with beta-catenin, as well as the *c-Myc* oncogene through direct binding to the promoter region of this gene [96–100]. Intriguingly, parafibromin also seems to exhibit oncogenic features in the presence of certain molecular partners, suggesting a yin-yang modus operandi of this protein [101].

In contrast to parathyroid adenomas, somatic *MEN1* mutations are very infrequent in parathyroid carcinomas, but nonetheless reported [102]. Given the exceedingly low rate of malignant PHPT in MEN1 kindred, other genetic events apart from this aberrancy are expected to drive the invasive behavior in parathyroid carcinoma.

*TP53* gene mutations are among the most common genetic aberrations in malignant epithelial tumors, although these genetic events seem to be unusual in parathyroid carcinomas [103–105]. Even so, LOH of one *TP53* allele seems to be more common [103]. Similarly, loss of the *retinoblastoma 1* (*RB1*) gene, encoding pRB, is observed in the majority of parathyroid carcinomas, however inactivating mutations have not been reported [106–108]. The P53 and pRB proteins are regulators of cell cycle progression and two bona fide tumor suppressors usually required to be silenced on both alleles in order to promote neoplasia, and given the fact that parathyroid carcinomas recurrently exhibit absent pRB expression, additional inactivating mechanisms apart from mutations are most likely operational [106].

Next-generation sequencing studies on parathyroid carcinomas are rare, not surprising given the low prevalence of this disease in general. In a recently published whole-genome sequencing study of 23 parathyroid carcinomas, the authors conclude that *CDC73* gene mutations were the most common sequence aberration, occurring in almost 40% of cases. Increased copy number variants were seen in parathyroid carcinomas with *CDC73* mutations, and these cases also carried an increased tumor mutational burden and poorer patient outcome. In unrelated, exome-based studies, recurrent mutations in *AarF domain containing kinase 1* (*ADCK1*) and *prune homolog 2 with BCH domain* (*PRUNE2*) have been reported in parathyroid carcinomas; however, their functional roles has not been elucidated [109, 110]. Moreover, a general overrepresentation of mutations in genes associated with DNA repair and cell cycle regulation seems evident [111, 112], and rare mutations in established cancer-associated genes have also been identified, for example *succinate dehydrogenase complex flavoprotein subunit A* (*SDHA*) and *DICER1*. From a therapeutic perspective, the majority of parathyroid carcinomas might carry alterations suitable for molecular targeted therapies, thus highlighting the potential role for next-generation sequencing as a tool to identify cases with potential for targeted therapeutic interventions [113].

Promoter mutations of the *telomerase reverse transcriptase* (*TERT*) gene are heavily implicated in human cancers, as they convey increased *TERT* expression, which in turn promotes immortalization. However, these mutations seem fairly rare in parathyroid carcinomas, although these tumors in general express TERT protein [112, 114, 115]. As of this, alterative mechanisms leading to increased TERT expression in parathyroid carcinomas are suspected.

Aberrant epigenetic mechanisms are also at play in parathyroid carcinomas. Via global methylome analyses, parathyroid carcinomas and adenomas seem to exhibit hypermethylation of *CDKN2B*, *CDKN2A*, *WT1*, *SFRP1*, *SFRP2*, and *SFRP4* [59]. Moreover, beside the recurrent *CDC73* promoter hypermethylation discussed above, altered methylation levels of the *adenomatous polyposis coli* (APC) promoter 1A region is recurrently seen in parathyroid tumors, although the *APC* mRNA expression seem to be retained by an unmethylated 1B promoter region [75, 116]. In parathyroid carcinomas specifically, loss of APC protein expression is a frequent event, and this could in part be due to aberrant methylation patterns rather than gene mutations [116–118]. The APC protein is a tumor suppressor that regulates the Wnt pathway, and loss of APC expression is therefore thought to stimulate proliferation in parathyroid cells. In more recent years, the regulation of epigenetic de-methylation has been highlighted in cancer, especially the discovery of the TET1/TET2 enzymatic activity catalyzing oxidation of 5-methylcytosine (5mC) to generate 5-hydroxymethylcytosine (5hmC). In parathyroid carcinomas, 5hmC levels and TET1 expression have been found extensively reduced, suggesting a general reduction in de-methylation events across the genome [119, 120]. As this phenomenon is tightly linked to the presence of *TERT* promoter mutations in unrelated cancer types, the general absence of these mutations in parathyroid carcinoma would suggest that other molecular mechanisms influence the observed lack of global de-methylation—a subject worthy of follow-up studies [121].

## The Diagnostic Challenges

### MEN1-Related PHPT: Could the Pathologist be of any Help?

The MEN1 syndrome is by far the most common among the hereditary conditions detailed in this review, with one case per 40,000 as opposed to MEN2A (1 case per 2 million) and the HPT-JT syndrome (unknown prevalence, but expected to be exceedingly low) [8]. Therefore, surgical pathologists will most likely diagnose MEN1 related parathyroid adenomas to a much larger extent than MEN2A, HPT-JT, FIHP, or MEN1-like related cases. Although the MEN1 syndrome often is diagnosed long before the patient is subjected to parathyroidectomy, the non-total penetrance in younger years as well as the occurrence of de novo* MEN1* mutations in subsets of patients with healthy parents allow for subsets of patients being misclassified as sporadic PHPT patients. As up to 10% of individuals with primary parathyroid “hyperplasia” (multiglandular disease, adenomatosis, multiple adenomas) have MEN1, genetic screening for familial disease has been considered for all patients with primary parathyroid “hyperplasia.” From a diagnostic pathology standpoint, is there a way for the attentive pathologist to aid in the detection of syndromic PHPT in cases where the syndromic association was not evident preoperatively?

*MEN1* was early on assigned a tumor-suppressor gene status, as the bulk of mutational events in MEN1 kindred reported represent inactivating events leading to premature truncations of the menin protein [122]. Moreover, most parathyroid adenomas arising in MEN1 patients harbor loss of heterozygosity (LOH) of the *MEN1* wild-type allele on the somatic level, thereby arguing in favor for the Knudson’s “two hit” theory in which bi-allelic inactivation of a tumor suppressor gene is needed in order for a tumor to develop [123–125]. Therefore, the question arises if clinical screening using expressional analyses targeting menin could be a cheap and efficient way for the surgical pathologist to triage menin-negative PHPT cases for genetic screening—as loss of menin expression would be indicative of *MEN1* gene aberrancies.

Menin is a predominantly nuclear protein with a highly conserved sequence among species, and the protein is universally expressed in most human tissues [122]. The protein harbors nuclear localization signals and leucine zipper motifs, both of which are features needed to regulate gene expression though direct interaction with DNA elements. For example, menin has been shown to interact with *JunD*, a proto-oncogene encoding a transcription factor regulating apoptosis and *TP53* gene activation (Fig. 2) [126]. Moreover, menin has been proposed as an important player in the regulation of various signaling networks associated to tumor development, such as the TGF-beta, RAS, Wingless type (Wnt) pathways—as well as influencing the expression of the telomerase reverse transcriptase (*TERT*) gene [127–130].

From a histological context, the MEN1-related parathyroid disease is often multiglandular, although not always synchronous in presentation [8]. Unlike sporadic parathyroid adenomas, MEN1-related tumors are often devoid of a rim of normal parathyroid tissue, and they may be composed of chief cells arranged in compact, sheet-like formations [8, 131]. The tumors may exhibit fibrosis and have similar appearance as parathyroid hyperplastic lesions arising in the setting of chronic renal failure, and almost always lack invasive features. Overall, findings of multiglandular disease and absence of a normal parathyroid rim in a PHPT patient should raise the suspicion of the MEN1 syndrome; however, a “rim” can also be seen in up to 10% of multiglandular disease. In all, there is no histological feature that sets MEN1-related adenomas apart from sporadic ones.

Expression studies regarding menin and associations to underlying MEN1 mutational status in parathyroid tumors have been promising, as menin immunohistochemistry seem to exhibit high sensitivity to detect underlying *MEN1* mutations and/or gene deletions [132, 133]. Even so, the interpretation of menin immunohistochemistry is not straightforward, and the lack of comprehensive studies on the subject makes menin immunohistochemistry inappropriate for clinical routine screening purposes. Moreover, as the *MEN1* gene is frequently deleted and mutated also on the somatic level in sporadic parathyroid adenomas, additional factors are needed to bring into consideration before suspecting MEN1 syndrome–related PHPT from expressional analyses in the pathology laboratory. For example, the penetrance of PHPT in MEN1 patients reaches 95% at 50 years of age, and therefore, the diagnosis of MEN1 in older patients with multiglandular disease should not be completely overlooked—although the bar for considering an MEN1 diagnosis should be even lower in adolescent patients [37]. In all, a concerted teamwork between endocrine surgeons and pathologists covering the patient’s medical history, disease presentation, radiology, and histopathology is probably needed to properly identify all MEN1-related PHPT in the clinical setting.

### HPT-JT-Associated PHPT: Clues from the Histopathological Workup?

As HPT-JT patients carry germline *CDC73* gene mutations or deletions, it comes as no surprise that the prevalence of parathyroid carcinoma in this patient category is much higher (15–30%) than in unselected PHPT patient cohorts (< 1%). As parathyroid carcinoma is so uncommon, it has been suggested that individuals diagnosed with parathyroid carcinoma should be promptly evaluated for HPT-JT. Additionally, if the pathologist raises the suspicion that the patient is indeed an HPT-JT kindred, a raised awareness of the increased parathyroid carcinoma risk is mandated when diagnosing the tumor. Easily recognizable features such as a tumor being cystic may in itself raise the possibility of HPT-JT syndrome. In the most comprehensive study yet regarding morphological features of *CDC73* mutated parathyroid tumors, the authors conclude that these lesions are characterized by sheet-like, compact growth rather than the usual acinar patterns visualized in the bulk of parathyroid tumors, as well as a typical eosinophilic cytoplasm distinct from the granular oxyphilic cell type often observed in areas of parathyroid adenomas [89]. Many tumors also exhibited enlarged nuclei and a perinuclear cytoplasmic clearing. In all, these findings suggest that the occurrence of these histological parameters might suggest the occurrence of an underlying *CDC73* gene mutation, and these cases should preferably be investigated more thoroughly with parafibromin immunohistochemistry and/or *CDC73* gene sequencing.

### Pinpointing Parathyroid Carcinoma in the Diagnostic Setting

Parathyroid carcinoma is the malignant form of PHPT. It constitutes only 0.5–3% of PHPT cases. As many parathyroid tumors present with various degrees of atypia, although not fulfilling the current WHO criteria for parathyroid carcinoma, there is a great need to identify malignant potential in these lesions by other means than histology alone. Moreover, any clinical marker of value in this context would need to exhibit very high specificity, in order to avoid falsely diagnosing benign tumors as carcinoma. The bulk of studies on this subject have attempted to address this dilemma via immunohistochemistry, which is a gold standard, inexpensive methodology used in most pathology laboratories and therefore suitable for rapid implementation in routine clinical practice [27].

One of the earliest immunohistochemical markers proposed to identify parathyroid carcinoma was Ki-67, an extensively used proliferation marker used to identify cells in active (non-G0) phases of the cell cycle. Although there seem to be an overrepresentation of carcinomas among high-proliferative parathyroid tumors, the overlap between parathyroid adenomas and carcinomas is considerable [134–139]. In a similar fashion, investigations of the cell cycle protein cyclin D1 was initially proven of value, as it was found upregulated in most parathyroid carcinoma compared to adenomas [140]. However, subsequent studies have identified overlap in expression between benign and malignant groups [117, 141].

Detection of widespread LOH of the *RB1* locus in parathyroid carcinoma with loss of its corresponding protein pRB prompted the investigation of pRB expression in parathyroid tumors—but with divergent results both concerning staining outcomes as well as LOH events [108, 134, 135, 142, 143]. In short, while LOH of the *RB1* locus seem to confer high sensitivity for the detection of parathyroid carcinoma, a significant amount of adenomas harbor the same genetic aberrancy, and the reduced specificity renders this marker of less value when screening for rare carcinomas in PHPT. Moreover, a positive pRb staining seems to be variable in terms of the proportion tumor cells stained, making the interpretation somewhat challenging [108].

Following the advent of tissue microarrays, a study concluded that the combined positivity of p27, B-cell lymphoma 2 (bcl-2), and mouse double minute 2 homolog (mdm2) adjoined by a low Ki-67 proliferation index indicated a benign clinical behavior of any given parathyroid tumor, as this profile was not evident in any parathyroid carcinoma but found in the vast majority of adenomas [136]. Both bcl-2 and mdm2 are members of the P53 pathway, thereby furthermore solidifying the relationship between aberrant P53 signaling and parathyroid carcinoma. However, follow-up studies have demonstrated overlaps between parathyroid adenomas and carcinomas, not least the finding of down-regulation of p27 in large subsets of parathyroid adenomas [63, 144–146]. P53 immunoreactivity shows variable staining in benign and malignant parathyroid tumors and does not appear to be useful as a single marker in separating these tumors [144, 144, 147]. Other diagnostic nomograms have been evaluated to differentiate malignant from benign parathyroid neoplasms, including among others protein gene product 9.5 (PGP 9.5), Ki-67, galectin-3, E-cadherin, and pRb markers [148]. More novel combinations of immunohistochemical and in situ hybridization approaches have also been evaluated, such as long noncoding RNA expression [149].

The most well-studied and reproduced marker in the context of distinguishing parathyroid carcinoma from adenoma is parafibromin, the 531 amino acid protein product of the *CDC73* main transcript [35]. Given the increased parathyroid carcinoma risk in HPT-JT kindred as well as the association between somatic *CDC73* mutations and sporadic parathyroid carcinoma, subsequent immunohistochemical analyses regarding parafibromin early-on proved of value to detect the majority of *CDC73* mutated cases, including most parathyroid carcinomas [90, 91, 93–95, 118]. Moreover, the vast majority of parathyroid adenomas analyzed have been shown to retain parafibromin immunoreactivity, apart from subsets of sporadic cases with a predominant cystic growth pattern, HPT-JT-associated parathyroid adenomas, and atypical parathyroid tumors [17, 57, 93, 150]. Thus, with the exceptions of the previously mentioned exceptions, parafibromin is useful for screening purposes in the PHPT population and can be a highly useful marker of malignant potential in many cases. The value of this marker in the clinical setting has been assessed both by comprehensive meta-analyses and by recent reports from high-volume centers [89, 92, 151, 151].

**Fig. 3:**
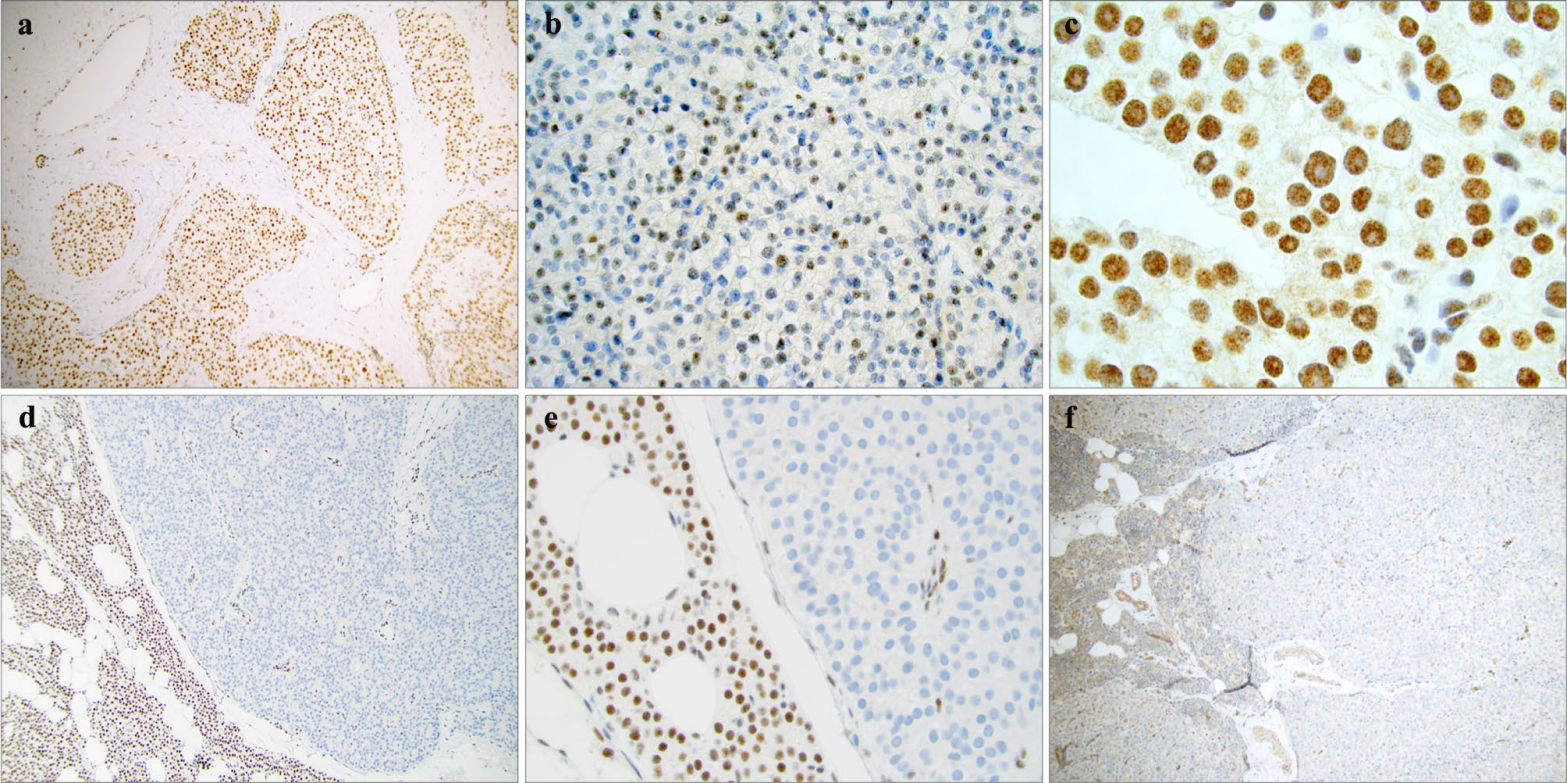
Examples of parafibromin and APC immunohistochemical staining patterns. **a** Diffusely positive nuclear parafibromin stain in an atypical parathyroid tumor with marked fibrosis. Magnification × 100. **b** Subsets of parathyroid tumors might present with a reduction in the proportion of parafibromin-positive cells, as depicted in this atypical tumor. Magnification × 400. **c** Absent nucleolar parafibromin expression is coupled to *CDC73* gene mutations affecting the regions encoding the nucleolar localization signals. Magnification × 1000. **d**, **e** Complete loss of parafibromin immunoreactivity in an atypical tumor, magnified × 100 and × 400, respectively. Note the positive nuclear staining in the adjacent normal rim of parathyroid tissue, as well as in endothelial cells within the tumor stroma. **f** Absent cytosolic APC immunoreactivity in an atypical parathyroid tumor. The normal parathyroid remnant (left) as well as the endothelial component within the tumor stain positive. Magnification × 100

As parafibromin is a predominant nuclear protein, loss of nuclear immunoreactivity is considered pathognomonic for an underlying inactivating *CDC73* mutation. As most *CDC73* mutations, be it germline or somatic, are either deleterious, located in early exons (causing premature termination) or missense alterations in important regulatory regions (such as the nuclear localization regions), mutated parafibromin is usually unable to reach its nuclear localization [97, 151, 152]. Most studies therefore seem to agree that almost all parathyroid tumors with wild-type *CDC73* sequence display a diffuse positive parafibromin stain (Fig. 3a), while reduced or absent nuclear parafibromin expression is an aberrant staining pattern and might signal the presence of a mutation (Fig. 3b, d, e). While diffuse loss of parafibromin expression is usually seen, reduced (but not complete) nuclear parafibromin expression can also be noted in *CDC73* mutated cases, occurring in subsets of tumor nuclei in a chessboard type of pattern (Fig. 3b) [92, 94]. Additionally, rare cases with parafibromin-negative nucleolar compartments have also been reported, which could be of importance given the nucleolar roles of wild-type parafibromin [88, 153–157]. Specifically, subsets of parathyroid tumors with *CDC73* mutations thought to disrupt the nucleolar localization signals might exhibit retained nuclear parafibromin immunoreactivity while evidently displaying negative nucleolar staining—and practicing pathologist should therefore be aware of this staining pattern as well (Fig. 3c) [156]. As most studies focus on the 2H1 monoclonal parafibromin antibody targeting the N-terminal, the usage of different antibodies should not explain these different staining patterns. Indeed, the “partial loss” pattern described above has been reported in cases assessed with four different parafibromin antibodies, suggesting that these observations are not biased by the selection of a specific target epitope [94]. The interpretation of parafibromin immunohistochemistry is therefore not completely straightforward, and additionally, the laboratory processing (including different antigen retrieval techniques and primary antibody incubation time) might affect the overall staining outcome [94, 131]. Moreover, parafibromin expression is often found stronger in the tumor periphery than in central aspects of the lesion, and therefore internal controls (such as endothelial cells) should be assessed and noted as positive before a negative parafibromin stain is called out (Fig. 3d, e) [93, 131].

**Fig. 4:**
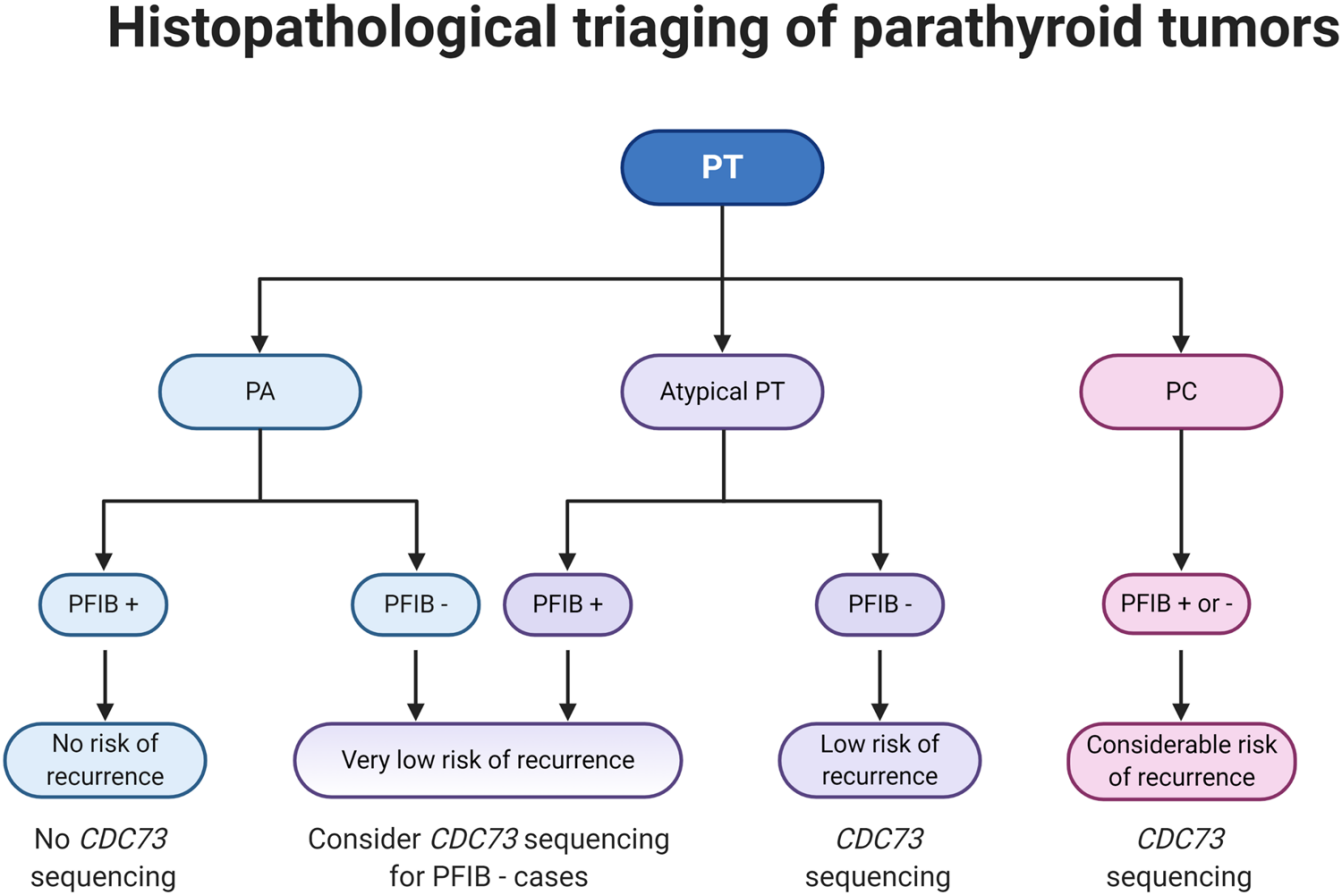
A proposed histopathological triaging scheme of a parathyroid tumor (PT) using immunohistochemistry (IHC). Parathyroid adenoma (PA) are rarely subjected to parafibromin (PFIB) immunohistochemistry, as these tumors almost always stain positive (+). However, subsets of cases with a cystic phenotype or with a known coupling to the hyperparathyroidism-jaw tumor (HPT-JT) or familial isolated hyperparathyroidism (FIHP) syndromes might be observed with reduced or negative immunoreactivity (−), and PFIB IHC might help triage cases for *CDC73* gene sequencing. An atypical parathyroid tumor (atypical PT) displays some histological features of PC without fulfilling the WHO criteria for PC (invasive growth). If positive for PFIB, these tumors have a low risk of recurrence, while PFIB negative cases have a non-negligible risk worthy of follow-up and should be interrogated for *CDC73* gene mutations (somatic and germline). PCs should probably be *CDC73* gene sequenced irrespectively of PFIB IHC outcome, as the sensitivity to detect *CDC73* gene mutations with PFIB IHC is non-perfect. Created with BioRender.com.

Most parathyroid tumors will be assessed for parafibromin immunoreactivity if they are atypical, i.e., lack undisputable evidence of malignancy but display worrisome features (be it clinical or histological) often seen in parathyroid carcinomas. The preponderance of these tumors stain positive, although large subsets can be parafibromin deficient [17, 91, 94, 150]. How should these cases be interpreted, and what should be reported to the surgeon? Consulting the literature, it seems evident the vast majority parafibromin-negative atypical tumors will behave benign, even after long-term follow-up [17, 91]. Even so, the recurrence rate is not entirely negligible, and the finding of an aberrant parafibromin stain should also highlight the need to exclude an underlying germline *CDC73* gene mutation—especially if there is a positive family history or if the proband itself exhibits multiglandular disease (Fig. 4) [17, 89, 91].

Given the non-perfect parafibromin sensitivity and specificity, researchers have been looking for additional markers to complement parafibromin immunohistochemistry. Shortly after parafibromin was coupled to the Wnt pathway, investigations of Wnt regulators detected widespread loss of APC immunoreactivity in most parathyroid carcinomas—which has later been coupled to promoter hypermethylation in PCs (Fig. 3f) [116, 117, 150]. As parathyroid adenomas are APC positive in general, the marker has gained ground as a clinical adjunct to parafibromin [117, 118]. However, as panels with two negative markers have its limitations in terms of interpretation associated to tissue fixation, researchers have looked for markers upregulated in parathyroid carcinomas. In this aspect, galectin-3 and PGP9.5 have both been shown to stain positive in the majority of parathyroid carcinomas while only expressed in few adenomas, and different panel combinations using galectin-3 and/or PGP9.5 with parafibromin have proven more reliable than using parafibromin alone [158-161].

Overall, the parathyroid carcinoma diagnosis is reserved for cases exhibiting unequivocal histological signs of invasive behavior, and no single molecular marker has yet been able to safely predict the malignant potential in atypical cases—which is most likely influenced by the general abundance of atypia in benign tumors and the rarity of truly metastatic cases. Moreover, there is also a potential sampling bias, as malignant tumors are not primarily resected if advanced disease is present at the time of diagnosis, and the fact that tumors with a molecular potential for spread might be resected well before their dissemination.

## Discussion

The molecular background of parathyroid neoplasia is well characterized from a driver gene perspective, not least due to the identification of mutations in genes responsible for the development of hereditary PHPT. These genes were subsequently found aberrant also in sporadic cases, and still—some 20 years after original identification—enjoy their position as the top recurrently mutated genes in parathyroid tumors. Modern molecular analyses have since then expanded our knowledge regarding common dysregulations in parathyroid tumors, and highlighted both genetic and epigenetic changes involved in the development of PHPT. Next-generation sequencing analyses of both parathyroid adenoma and carcinoma cohorts have helped identify additional genes of potential impact for both groups, although it is clearly demonstrated that the “low-hanging fruit” in terms of highly recurrent events already have been picked.

From a clinical perspective, *CDC73* mutations and loss of parafibromin expression have been firmly established as events coupled to parathyroid malignancy. Additionally, comprehensive sequencing of parathyroid carcinoma has identified mutations suitable for molecular targeted therapy as well as overall mutational burden as a prognostic tool of potential value, thereby highlighting the usage of modern genetic analyses where histology and immunohistochemistry might be insufficient. Given the rapid evolution of molecular techniques used for complimentary analyses in the routine clinical setting, the combination of histology, immunohistochemistry and next-generation sequencing might constitute standard work-up for parathyroid tumors displaying significant atypia in the near future.

To summarize the field for the surgical pathologist, parathyroid adenoma is usually a straightforward diagnosis, but there are histological patterns coupled to underlying syndromes worth remembering—as they could help identify hereditary disease of importance for clinical follow-up. When present, histologically atypical features in a parathyroid lesion could signify malignant potential, but invasive growth is still the only accepted criteria to diagnose parathyroid carcinoma. A plethora of immunohistochemical markers could potentially aid in the identification of parathyroid carcinoma, but many display subpar specificity and should be interpreted with caution. Loss of parafibromin nuclear expression correlates with the presence of an underlying *CDC7*3 gene mutation, which in turn is coupled to an increased risk of developing parathyroid carcinoma. The mutation might also be present in germline tissues and thus predispose to familial disease.

Even though much of the genetic landscape of parathyroid tumors has been studied, two key queries remain to be deciphered, namely (1) the identification of additional driver genes responsible for the development of parathyroid adenomas, and (2) the quest for additional molecular events driving the malignant potential of parathyroid tumors besides parafibromin. As approximately half of parathyroid adenomas lack mutations in the most common driver genes, additional genetic events are expected to be found. Moreover, as the majority of HPT-JT kindred develop adenomas, other genetic aberrations apart from the inactivation of *CDC73* are likely to be required to propel the invasive behavior in parathyroid carcinomas. Hopefully, these questions can be solved in the future by comprehensive, next-generation sequencing studies using multi-center tumor cohorts.
